# Supplementary material for: Species composition and environmental adaptation of indigenous Chinese cattle
Source: Sci Rep. 2017 Nov 23;7:16196. doi: 10.1038/s41598-017-16438-7 (PMC5700937; doi:10.1038/s41598-017-16438-7)
Supplement: Supplementary file 1 — Supplementary Figures S1-S6 and Tables S1, S2, S5, S6. [file 41598_2017_16438_MOESM1_ESM.pdf]

Species composition and environmental adaptation of indigenous Chinese cattle

Yahui Gao[1], Mathieu Gautier[2,3], Xiangdong Ding[1], Hao Zhang[1], Yachun Wang[1], Xi Wang[4], MD Omar Faruque[5], Junya Li[6], Shaohui Ye[7], Xiao Gou[7], Jianlin Han[8,9], Johannes A. Lenstra[10], Yi Zhang[1]*

[1] National Engineering Laboratory for Animal Breeding, Key Laboratory of Animal Genetics and Breeding and Reproduction of MOA, College of Animal Science and Technology, China Agricultural University, Beijing 100193, China.

[2] INRA, UMR CBGP (INRA-IRD-Cirad-Montpellier SupAgro), Campus international de Baillarguet, Montferrier-sur-Lez, France.

[3] Institut de Biologie Computationnelle, 95 rue de la Galera, 34095 Montpellier, France.

[4] Institute of Animal Science and Veterinary Medicine, Shanxi Academy of Agricultural Science, Taiyuan 030032, China.

[5] Department of Animal Breeding and Genetics, Bangladesh Agricultural University, Mymensingh-2202, Bangladesh.

[6] Institute of Animal Science, Chinese Academy of Agricultural Sciences, Beijing 100193, China.

[7] College of Animal Science and Technology, Yunnan Agricultural University, Kunming 650201, China.

[8] CAAS-ILRI Joint Laboratory on Livestock and Forage Genetic Resources, Institute of Animal Science, Chinese Academy of Agricultural Sciences (CAAS), Beijing 100193, China.

[9] ILRI International Livestock Research Institute (ILRI). P.O. Box 30709, Nairobi 00100, Kenya.

[10] Faculty of Veterinary Medicine, Utrecht University, Yalelaan 104, 3584 CM Utrecht, The Netherlands.

*Corresponding author

Email: yizhang@cau.edu.cn

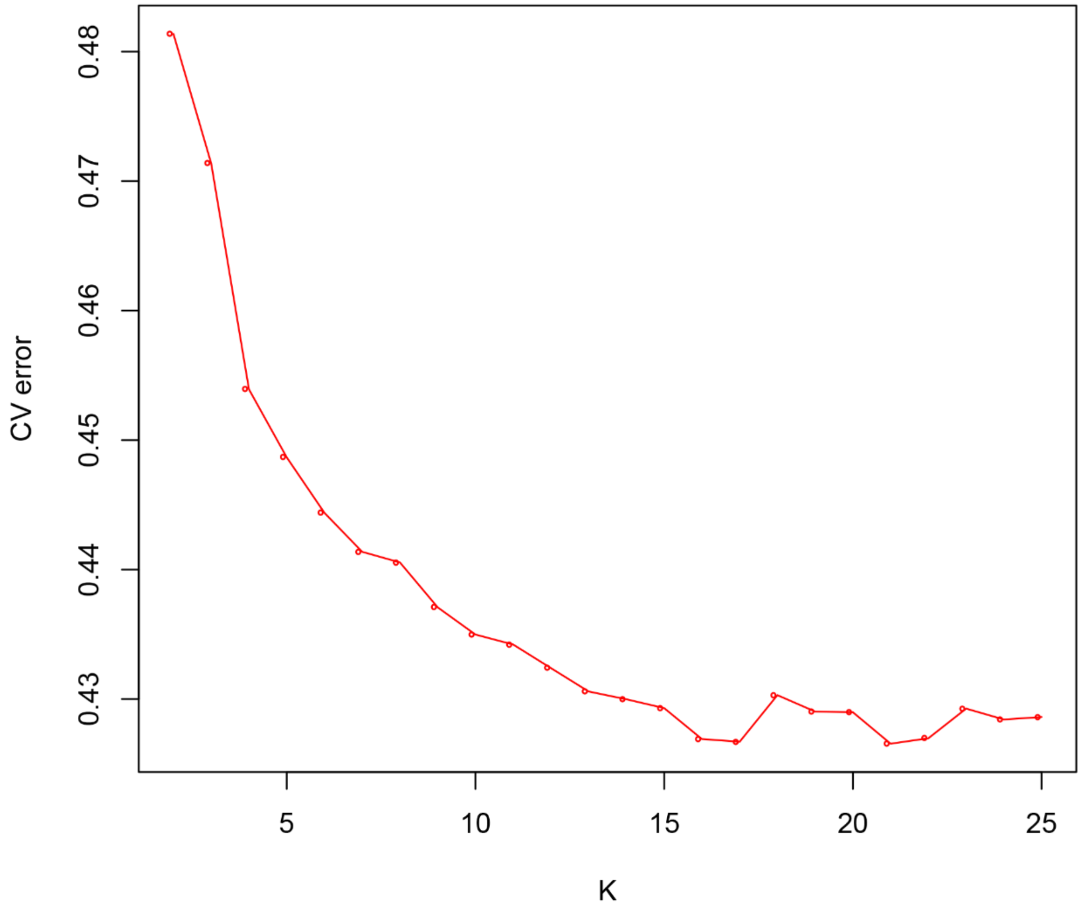

Figure S1. Cross validation in admixture analysis to find the best K.

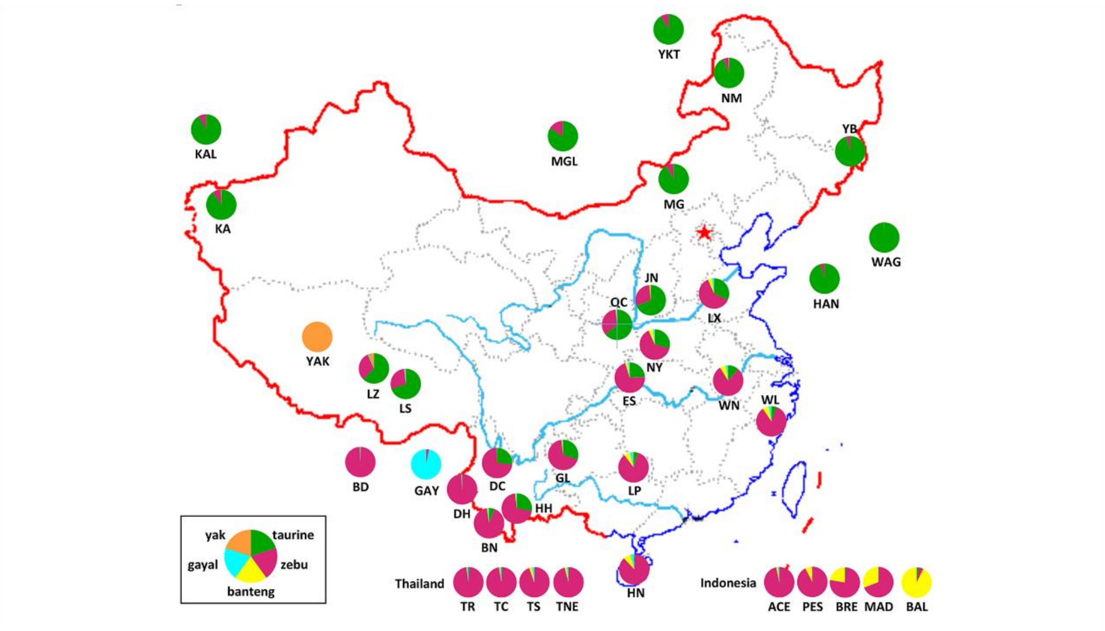

Figure S2. Geographical plot of genetic components on map. It was modified with Microsoft PowerPoint from a blank map of China available from ChinaMaps.org (http://www.chinamaps.org).

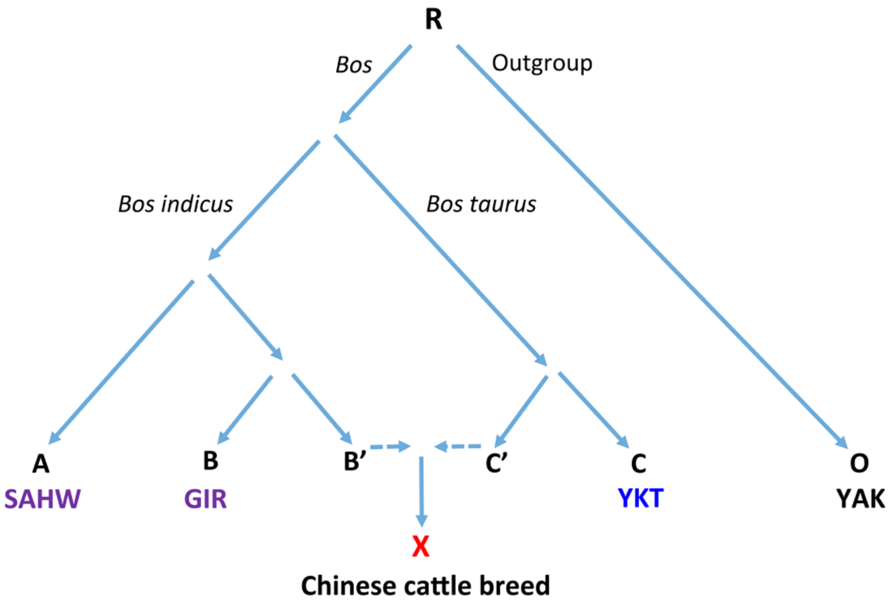

Figure S3. Phylogeny to estimate the proportion of indicine ancestry in Chinese cattle populations based on *f4* ratio test.

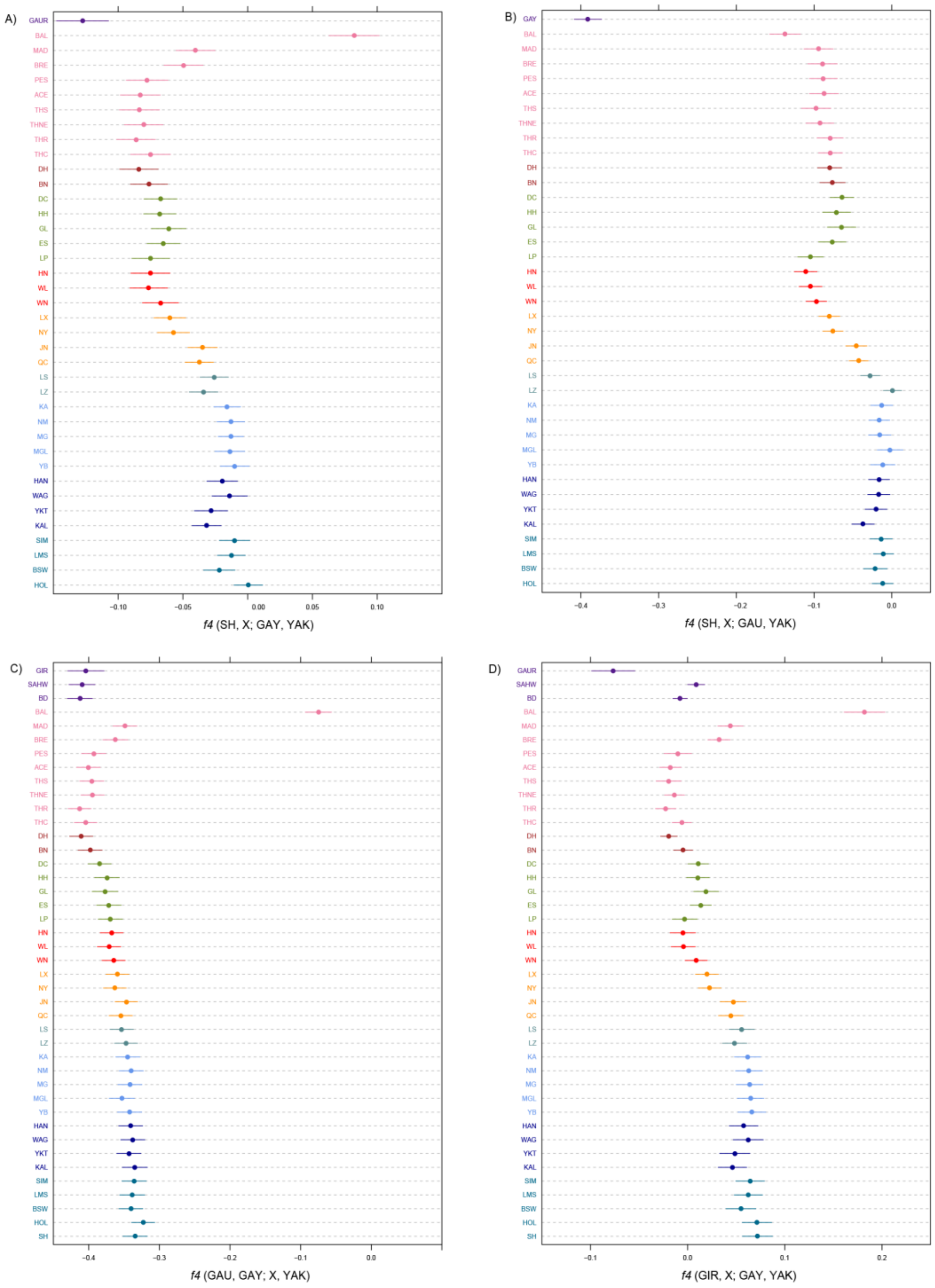

Figure S4. *f4*-statistics to test gene flow. (A) *f4*-statistics of the form D (SH, X; GAY, YAK). (B) *f4*-statistics of the form D (SH, X; GAU, YAK). (C) *f4*-statistics of the form D (GAU, GAY; X, YAK). (D) *f4*-statistics of the form D (GIR, X; GAY, YAK). The whiskers represent the standard error.

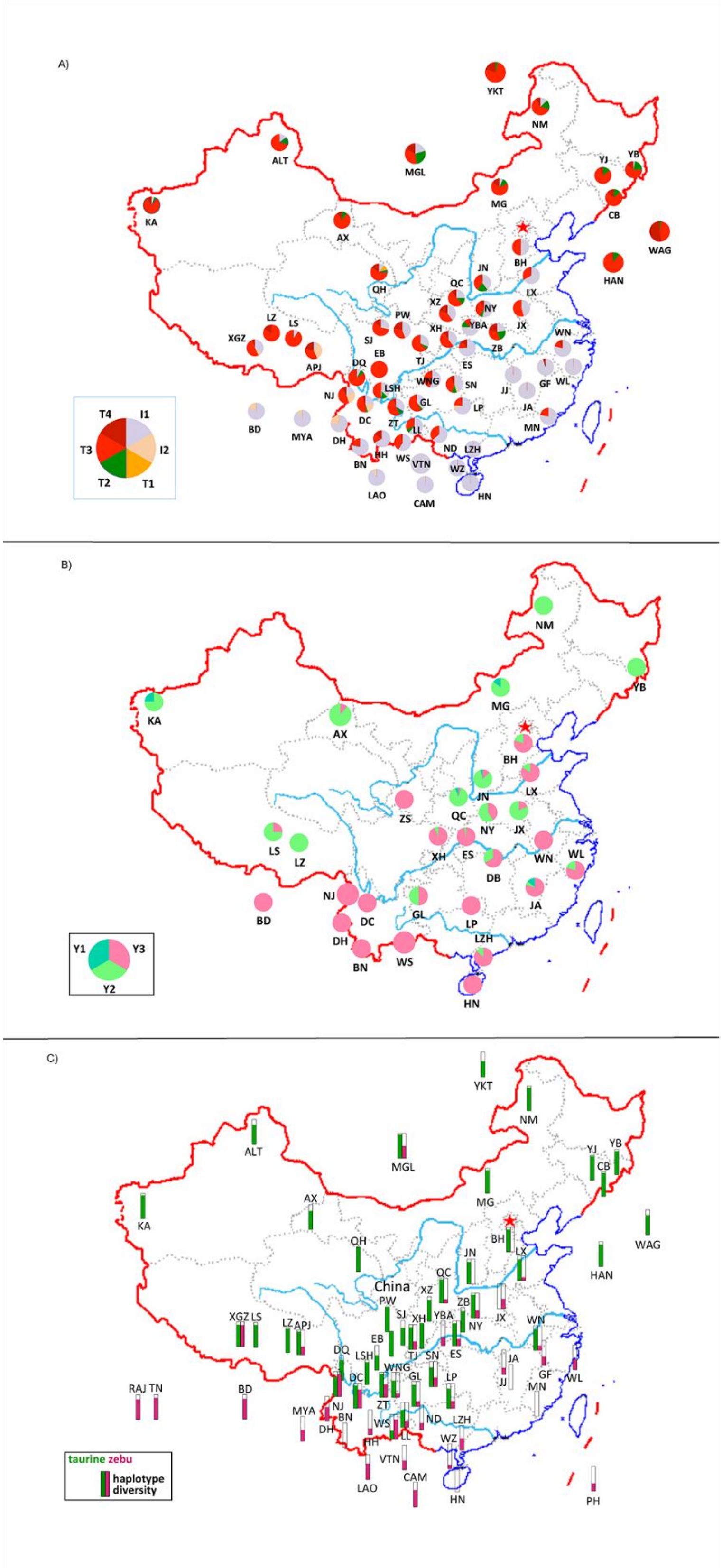

Figure S5. Distribution of mtDNA haplogroups and Y chromosome types in Chinese cattle. (A) mtDNA haplogroups in Chinese cattle. Data are shown in Table S3. (B) Y chromosome types in Chinese cattle. Data are shown in Table S3. (C) mtDNA haplotype diversity of taurine and indicine types in Chinese cattle populations. Data are shown in Table S3. Map was modified with Microsoft

PowerPoint from a blank map of China available from ChinaMaps.org (http://www.chinamaps.org).

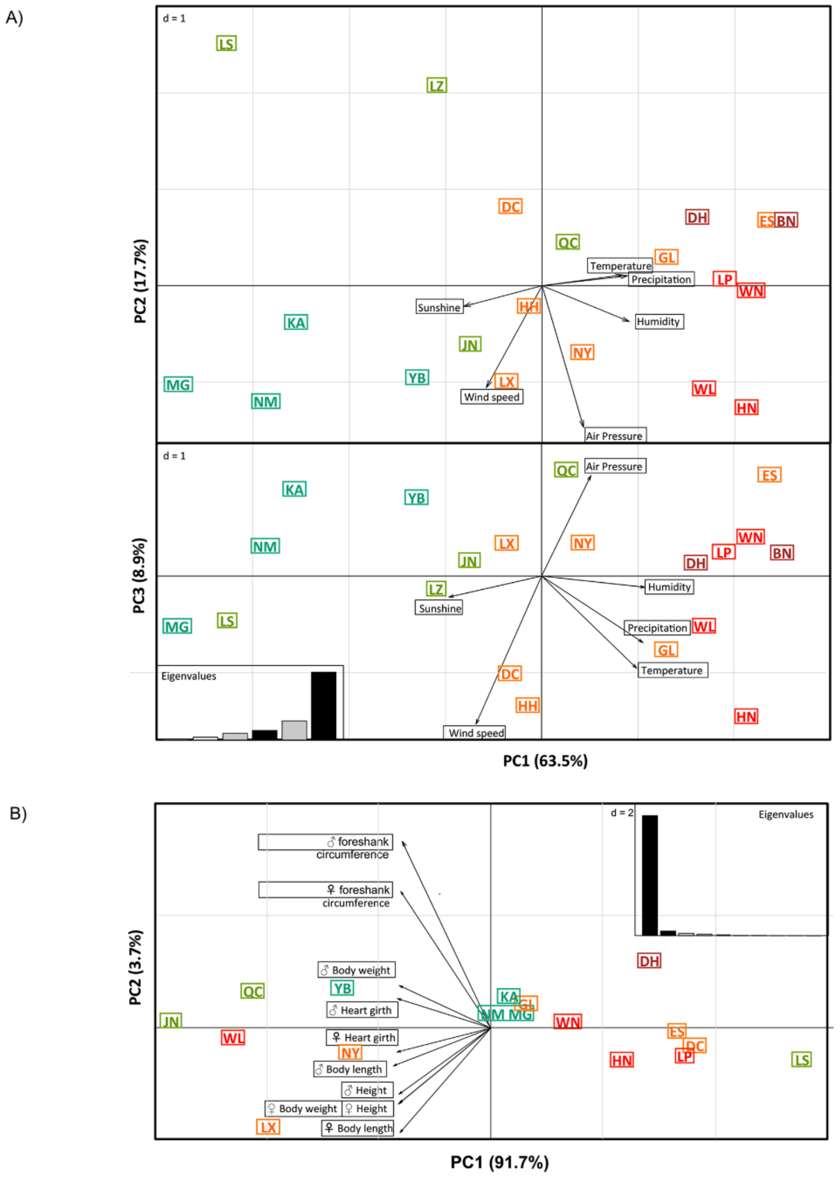

Figure S6. PCA on various population specific environmental and morphological covariables. (A) PCA on the six scaled environmental covariables. (B) PCA on the scaled morphological covariables.

Population		*f4* ratio estimation			Admixture membership of taurine genome	
Region	Population	α	SD	Z(null=0)	α	SD
N China	KA	1.041	0.008	123.24	0.906	0.015
N China	MG	1.066	0.008	128.65	0.918	0.021
N China	NM	1.065	0.009	124.86	0.936	0.014
Tibet	LZ	0.656	0.007	90.28	0.625	0.100
Tibet	LS	0.770	0.007	103.93	0.696	0.083
Central China	QC	0.693	0.007	93.18	0.639	0.015
Central China	JN	0.760	0.008	90.15	0.691	0.022
Central China	NY	0.282	0.007	39.71	0.291	0.022
Central China	LX	0.315	0.008	41.27	0.323	0.027
SW China	ES	0.240	0.007	35.99	0.244	0.026
SW China	GL	0.320	0.010	33.52	0.300	0.065
SW China	HH	0.297	0.008	39.01	0.274	0.068
SW China	DC	0.297	0.006	46.65	0.265	0.023
SE China	WN	0.065	0.008	7.81	0.116	0.058
SE China	WL	0.008	0.009	0.84	0.060	0.082
SE China	HN	-0.109	0.009	-11.60	0.009	0.017
SE China	LP	-0.027	0.010	-2.78	0.051	0.016
Far-SW China	BN	0.055	0.006	8.68	0.061	0.041
Far-SW China	DH	-0.016	0.005	-3.00	0.006	0.024

Supplementary Table S1. Proportion of taurine ancestry in Chinese cattle populations estimated by *f4* ratio test and admixture analysis. Referring to the phylogeny of Fig. S2, *f4* ratio estimation was conducted based on the assumption that Chinese cattle population (X) is an admixture of Asian zebu (B) and taurine cattle (C). The admixture membership of taurine was obtained from a supervised admixture analysis when K=5 (Fig. 3b).

Country/Region	Breed	Code	mtDNA												Y chromosome				
				**mtDNA haplogroup**							**Taurine**		**Indicine**			**Y haplotype**			
				**N**	**T1**	**T2**	**T3**	**T4**	**I1**	**I2**	**N**	**Hd**	**N**	**Hd**		**N**	**Y1**	**Y2**	**Y3**
NW China	Anxi	AX	Lai et al. (2006)	10		1	8	1			10	0.756			Li et al. (2013)	28		25	3
NW China	Altay	ALT	Jia et al. (2010)	14		2	10		2		12	0.788	2						
NW China	Kazak	KA	this study	16		1	13	1	1						this study	4	1	3	
			Jia et al. (2010)	1				1							Li et al. (2013)	5	2	3	
			Lei et al. (2006)	4			4												
			total	21		1	17	2	1		20	0.937	1		total	9	3	6	
N China	Mongolian XilinGol	MG	this study	30		2	18	8	2						this study	23	7	16	
			Jia et al. (2010)	8		2	6								Li et al. (2013)	25		25	
			Lei et al. (2006)	10		2	7		1										
			total	48		6	31	8	3		45	0.933	3		total	48	7	41	
N China	Mongolian HulunBuir	NM	this study	23		4	14	2	3		20	0.926	3		Li et al. (2013)	1		1	
N China	Changbai	CB	Jia et al. (2010)	15		2	11	2			15	0.943							
N China	Yanbian	YB	this study	25		4	15	5	1		36				this study	9		9	
			Jia et al. (2010)	2		1		1											
			Lei et al. (2006)	10		3	7												
			total	37		8	22	6	1			0.943	1		total	9		9	
N China	Yanjiang	YJ	Jia et al. (2010)	14		2	11	1			14	0.967							
Central China	Xizhen	XZ	Lei et al. (2006)	10			4	2	4		6	0.867	4						
Central China	Jinnan	JN	this study												this study	14	1	13	
			Lai et al. (2006)	4			3	1											
			Lei et al. (2006)	11		3	2		6						Li et al. (2013)	10		7	3

			total	14	3	5	1	6	9	0.889	6	0	total	24	1	20	3
Central China	Bohai	BH	Lei et al. (2006)	10		5		5	5	0.9	5	0		5		4	1
Central China	Luxi	LX	this study	11		3		8					this study	6		4	2
			Lei et al. (2006)	10		3	1	6					Li et al. (2013)	19			19
			total	21		6	1	14	7	0.857	14	0.143	total	25		4	21
Central China	Jiaxian	JX	Lei et al. (2006)	11		6		5	6	0	5	0.4		22		18	4
Central China	Nanyang	NY	this study	23	2	7	1	13					this study	6		1	5
			Lei et al. (2006)	11		4	2	5					Li et al. (2013)	31		21	10
			total	34	2	11	3	18	16	0.933	18	0.307	total	37		22	15
Central China	Qinchuan	QC	this study	29	2	15	2	10					this study	6		4	2
			Lai et al. (2006)	6	1	5											
			Lei et al. (2006)	13	3	5	3	2					Li et al. (2013)	47	3	41	3
			total	48	6	25	5	12	36	0.959	12	0.167	total	53	3	45	5
Central China	Zaobei	ZB	Lai et al. (2006)	9	2	3	2	2	7	0.857	2						
Central China	Yunba	YBA	Jia et al. (2010)	8	1	1		6	2		6	0.333					
S China	Wannan	WN	this study	30		3	1	26					this study	2			2
			Lai et al. (2006)	8		2	2	4					Li et al. (2013)	15			15
			total	38		5	3	30	8	0.857	30	0.193	total	17			17
S China	Minnan	MN	Lei et al. (2006)	9		2		7	2		7	0					
S China	Leizhou	LZH	Lei et al. (2006)	11				11			11	0.473	Li et al. (2013)	22		3	19
S China	Longlin	LL	Jia et al. (2010)	13	1	4		8	5	0.7	8	0.25					
S China	Nandan	ND	Jia et al. (2010)	11		4		7	4		7	0.286					
S China	Weizhou	WZ	Jia et al. (2010)	13				13			13	0.154					
S China	Hainan	HN	this study	4				4					this study	4			4
			Lei et al. (2006)	5				5					Li et al. (2013)	17			17
			total	9				9			9	0	total	21			21

S China	Guangfeng	GF	Jia et al. (2010)	21			1	20		1		20	0.358					
S China	Ji'an	JA	Lei et al. (2006)	6				6				6	0	Li et al. (2013)	14	2	1	11
S China	Jinjiang	JJ	Jia et al. (2010)	7				7				7	0.286					
S China	Wenling	WL	this study	29				29						this study	5		1	4
			Jia et al. (2010)	12				12										
			total	41				41				41	0.42					
S China	Enshi	ES	this study	30		3	4	23						this study	17			17
			Jia et al. (2010)	13		3	1	9						Li et al. (2013)	9		1	8
			total	43		6	5	32		11	0.909	32	0.288	total	26		1	25
S China	Ebian	EB	Lai et al. (2006)	5		5				5	0.6							
S China	Liangshan	LSH	Jia et al. (2010)	8	1	4		3		5	0.9	3						
SW China	Pingwu	PW	Jia et al. (2010)	9		3	2	4		5	1	4						
SW China	Sanjiang	SJ	Lai et al. (2006)	7		4	1	2		5	0.7	2						
SW China	Xuanhan	XH	Lei et al. (2006)	10		5	1	4		6	1	4		Li et al. (2013)	14		1	13
SW China	Tongjiang	TJ	Chen et al. (2008)	54	3	33	1	17		37	0.895	17	0.324					
SW China	Guanling	GL	this study	4		2		2						this study	4		2	2
			Lei et al. (2006)	22	1	12	1	8										
			total	26	1	14	1	10		16	0.858	10	0.2					
SW China	Liping	LP	this study	4				4						this study	5			5
			Lei et al. (2006)	21		4	2	13	2									
			total	25		4	2	17	2	6	0.8	19	0.292					
SW China	Sinan	SN	Lei et al. (2006)	22	2	8	2	9	1	12	0.773	10	0.378					
SW China	Weining	WNG	Lei et al. (2006)	22		9	1	12		10	0.667	12	0.167					
SW China	Diqing	DQ	Gou et al. (2010)	25	2	20	1	2		23	0.83	2						
SW China	Honghe	HH	this study	12		2	2	8		4		8	0.25					
SW China	Zhaotong	ZT	Jia et al. (2010)	21	2	11	1	7		14	0.945	7	0.524					

SW China	Dengchuan	DC	this study	31		1	13	3	8	6	17	0.912	14	0.747	this study	4		4
SW China	Wenshan	WS	Gou et al. (2010)	27			10	1	16		11	0.345	16	0.792	Gou et al. (2010)	16		16
Far-SW China	Nujiang	NJ	Gou et al. (2010)	11			5	1	2	3	6	0.8	5	0.9	Gou et al. (2010)	3		3
Far-SW China	Banna	BN	this study	14			3		11		3		11	0	this study	8		8
Far-SW China	Dehong	DH	this study	16					12	4			16	0.575	this study	5		5
Tibet China	Qinghai	QH	Shi et al. (2004)	19	2	1	11	3	2		17	0.993	2					
Tibet China	Apeijiaza	APJ	Jia et al. (2010)	12			5	1	1	5	6	0.933	6	0.333				
Tibet China	Linzhi	LZ	this study	19			18	1			19	0.971			this study	1	1	
Tibet China	Tibetan_Lasa	LS	this study	14			12		2						this study	4	3	1
			Jia et al. (2010)	7			7											
			total	21			19		2		19	0.912	2					
Tibet China	Xigaze	XGZ	Jia et al. (2010)	14			7	1	5	1	8	0.893	6	0.867				
Siberia Russia	Yakut	YKT	Kantanen et al. (2009)	24		1	18	5			24	0.645						
Japan	Japanese Black	WAG	Mannen et al. (1998)	32		1	14	17			32	0.794						
Korean	Hanwoo	HAN	Mannen et al. (2004)	30		3	26	1				0.892						
Mongolia	Mongolia	MGL	Mannen et al. (2004)	44		12	15	8	9		35	0.961	9	0.5				
VietNam	VietNam	VTN	Chen et al. (2010)	29					29				29	0.377				
Cambodia	Cambodia	CAM	Chen et al. (2010)	30					28	2			30	0.671				
Myanmar	Myanmar	MYA	Chen et al. (2010)	30					28	2			30	0.462				
Laos	Laos	LAO	Chen et al. (2010)	30					29	1			30	0.655				
Philippine	Philippine	PH	Komatsu et al. (2004)	18					18				18	0.307				
Bangladesh	Bangladesh	BD	this study	17					14	3			17	0.831	this study	13		13
India	Rajasthan	RAJ	Chen et al. (2010)	21					16	5			21	0.824				
India	Tamil Nadu	TN	Chen et al. (2010)	59					36	23			59	0.881				

Supplementary Table S2. mtDNA and Y-chromosomal variation.

Regions of this study	Regions overlapped with Gutiérrez-Gil et al. (2015)	Regions overlapped with Randhawa et al. (2016)	
		Based on meta-assembly of 4 groups of cattle [1]	Based on meta-assembly of 16 breeds of cattle
BTA02 : 61500000 - 62500000	CSS - 43 (BTA02: 61691551 - 62433744)	HS27 (BTA02: 59592000 - 64386000)	HS33 (BTA02: 59655000 - 62411000)
BTA03 : 33000000 - 34500000	CSS - 66 (BTA03: 33534426 - 33635160)	HS41 (BTA03: 33691000 - 35521000)	no
BTA03 : 46500000 - 48000000	CSS - 67 (BTA03: 38150000 - 47800000)	no	no
BTA03 : 90000000 - 91500000	CSS - 73 (BTA03: 89600000 - 99492000)	HS44 (BTA03: 90250000 - 97596000)	HS57 (BTA03: 87808000 - 103480000)
BTA04 : 30000000 - 31000000	no	HS48 (BTA04: 26677000 - 32969000)	HS63 (BTA04: 28855000 - 30372000)
BTA05 : 17500000 - 19000000	CSS - 103 (BTA05: 17902277 - 19669793)	HS58 (BTA05: 16907000 - 22679000)	HS77 (BTA05: 15063000 - 21822000)
BTA05 : 69500000 - 71000000	CSS - 110 (BTA05: 68294607 - 72549211)	HS63 (BTA05: 67598000 - 77732000)	HS83 (BTA05: 67598000 - 72294000)
BTA06 : 17500000 - 19000000	CSS - 117 (BTA06: 18592851 - 18605300)	HS71 (BTA06: 16563000 - 18323000)	HS90 (BTA06: 15891000 - 17759000)
BTA06 : 39500000 - 41500000	CSS - 123 (BTA06: 37355568 - 42446118)	HS73 (BTA06: 35633000 - 41259000)	HS92 (BTA06: 37192000 - 41259000)
BTA06 : 46000000 - 47500000	no	HS74 (BTA06: 46315000 - 53250000)	HS94 (BTA06: 45750000 - 46998000)
BTA06 : 73500000 - 75000000	CSS - 130 (BTA06: 67850000 - 83374529)	HS76 (BTA06: 68250000 - 74531000)	HS98 (BTA06: 66555000 - 73600000)<br>HS99 (BTA06: 74672000 - 77771000)
BTA06 : 105000000 - 106500000	CSS - 133 (BTA06: 105390830 - 105730372)	HS81 (BTA06: 103685000 - 111132000)	HS103 (BTA06: 103450000 - 110657000)
BTA08 : 59500000 - 60500000	no	HS101 (BTA08: 58282000 - 64077000)	HS125 (BTA08: 51250000 - 64202000)
BTA08 : 81000000 - 82000000	no	HS103 (BTA08: 79112000 - 83522000)	no
BTA08 : 111500000 - 113000000	no	no	HS135 (BTA08: 112737000 - 112737000)
BTA10 : 31000000 - 32500000	no	HS118 (BTA10: 27711000 - 32875000)	HS153 (BTA10: 27126000 - 32875000)
BTA11 : 9000000 - 10500000	no	HS127 (BTA11: 9343000 - 9665000)	HS165 (BTA11: 8205000 - 12905000)
BTA11 : 22500000 - 24500000	no	HS128 (BTA11: 20510000 - 28261000)	HS167 (BTA11: 19829000 - 24750000)
BTA12 : 34000000 - 35500000	no	no	no
BTA12 : 78000000 - 79000000	no	HS144 (BTA12: 71025000 - 78810000)	HS189 (BTA12: 76250000 - 79378000)
BTA13 : 45500000 - 48500000	CSS - 246 (BTA13: 46393167 - 48665879)	HS151 (BTA13: 44188000 - 52900000)	HS199 (BTA13: 45394000 - 49750000)
BTA13 : 50500000 - 52000000	no	HS151 (BTA13: 45394000 - 52900000)	HS200 (BTA13: 51632000 - 56204000)

BTA13 : 53000000 - 54500000	no	HS152 (BTA13: 53256000 - 56204000)	HS200 (BTA13: 51632000 - 56204000)
BTA15 : 52000000 - 53500000	CSS - 269 (BTA15: 51845122 - 52127428)	HS173 (BTA15: 49572000 - 58746000)	no
BTA20 : 67500000 - 69000000	no	HS214 (BTA20: 67162000 - 71982000)	no
BTA21 : 27500000 - 29000000	no	no	HS280 (BTA21: 27534000 - 29750000)
BTA24 : 39500000 - 40500000	no	HS237 (BTA24: 39908000 - 40305000)	no
1 HS represents for Hotspot ID			

Supplementary Table S5. Genomic regions identified this study overlap with previous reports.

Principal component	Temperature	Humidity	Sunshine	Air Pressure	Wind Speed	Precipitation
Environmental						
PC1	-0.870	-0.948	0.854	-0.450	0.604	-0.928
PC2	0.062	-0.206	-0.118	-0.812	-0.584	0.057
PC3	-0.316	-0.038	-0.072	0.344	-0.507	-0.228
Morphological						
PC1	0.085	-0.001	0.010	-0.508	-0.213	0.237

Supplementary Table S6. Correlation between scaled environmental and morphological principal components and original environmental factors.

Principal component	M. (F.) Weight	M. (F.) Height	M. (F.) Length	M.(F.) Heart Girth	M. (F.) Fore. Circ.
Environmental					
PC1	-0.006 (0.027)	-0.042 (0.077)	0.036 (0.081)	0.058 (0.079)	0.121 (0.036)
PC2	-0.454 (-0.504)	-0.455 (-0.464)	-0.584 (-0.55)	-0.530 (-0.533)	-0.472 (-0.449)
PC3	0.325 (0.218)	0.317 (0.323)	0.228 (0.10)	0.297 (0.198)	0.334 (0.331)
Morphological					
PC1	-0.956 (-0.966)	-0.961 (-0.956)	-0.973 (-0.95)	-0.976 (-0.981)	-0.916 (-0.941)

Supplementary Table S7. Correlation between (scaled) environmental and morphological principal components and original morphological traits.
